# The cardiometabolic conditions of psoriatic disease

**DOI:** 10.3389/fimmu.2022.970371

**Published:** 2022-09-08

**Authors:** Eric Toussirot, Irène Gallais-Sérézal, François Aubin

**Affiliations:** ^1^ INSERM CIC-1431, Centre d’Investigation Clinique, Pôle Recherche, CHU de Besançon, Besançon, France; ^2^ Rhumatologie, Pôle PACTE (Pathologies Aiguës Chroniques Transplantation Éducation), CHU de Besançon, Besançon, France; ^3^ Département Universitaire de Thérapeutique, Université de Franche-Comté, 25000 Besançon, France; ^4^ UMR 1098 RIGHT, INSERM, Établissement Français du Sang, Université Bourgogne Franche-Comté, Besançon, France; ^5^ Dermatologie, Pôle PACTE (Pathologies Aiguës Chroniques Transplantation Éducation), CHU de Besançon, Besançon, France

**Keywords:** psoriatic arthritis, cardiovascular risk, metabolic syndrome, psoriasis, obesity

## Abstract

Psoriasis (PsO) and psoriatic arthritis (PsA), together known as psoriatic disease (PsD), are immune-mediated diseases with a chronic and relapsing course that affect the skin, the joints or both. The pathophysiology of PsO is complex and involves abnormal expression of keratinocytes and infiltration of the skin with dendritic cells, macrophages, neutrophils and T lymphocytes. Around 30% of patients with PsO develop arthritis with axial and/or peripheral manifestations. Both PsO and PsA share similar Th1- and Th17-driven inflammation, with increased production of inflammatory cytokines, including TNFα, IFN-γ, IL-17, IL-22, IL-23 in the skin and the synovial membrane. PsD is associated with a high burden of cardiometabolic diseases such as hypertension, diabetes, dyslipidemia, obesity, metabolic syndrome and cardiovascular (CV) complications as compared to the general population. These comorbidities share common immunopathogenic pathways linked to systemic inflammation, and are associated with the extent and severity of the disease. Morever, they can influence treatment outcomes in PsD. In this short review, we summarize the available evidence on the epidemiology, clinical aspects and mechanisms of cardiometabolic conditions in patients with PsD. We also discuss the impact of targeted treatments such as methotrexate and biological agents on these cardiometabolic conditions.

## Introduction

Psoriasis (PsO) is a chronic skin disease affecting around 3% of the worldwide population. It is an immune-mediated disease characterized by abnormal expression of keratinocytes and infiltration of the dermis with dendritic cells, macrophages, neutrophils and T lymphocytes (1). Around 30% of patients with PsO develop psoriatic arthritis (PsA), a condition that is included in the spondyloarthritis group. Indeed, PsA may involve the axial skeleton and/or the peripheral joints, but also entheseal structures, overall leading to joint damage, physical limitation and disability (2). Both PsO and PsA share similar Th1- and Th17-driven inflammation, with increased production of inflammatory cytokines including TNFα, IFN-γ, IL-6, IL-8, IL-17, IL-23 in the skin and synovial membrane. Due to common pathophysiological mechanisms and complications, PsOs and PsA are collectively grouped under the term psoriatic disease (PsD) and are associated with specific extra-cutaneous/articular manifestations and comorbidities. Collaborative and multidisplinary approaches with dermatologists and rheumatologists have been developed in different countries in order to better diagnose and manage patients with PsD (3). There is compelling evidence that PsD carries a greater of developing cardiometabolic diseases as compared to the general population (4, 5). Higher cardiovascular (CV) risk is well documented in immune-mediated diseases such as rheumatoid arthritis (RA) or systemic lupus erythematosus (6). In parallel, there is a growing interest in CV and metabolic diseases in patients with PsD (4, 5, 7).

In this narrative review, we aim to analyse the cardiometabolic conditions of patients with PsD, with a focus on the underlying mechanisms. We also examine the influences that these comorbidities may have on treatment response in PsD patients, as well as the impact of conventional and targeted drugs on these conditions.

## Cardiometabolic comorbidities in PsD

### CV diseases

The association between CV mortality and morbidity is well described in patients with RA, and RA itself is considered to be an independant CV risk factor similar to the risk induced by type 2 diabetes (8). CV risk is also enhanced in PsA, and the prevalence of CV diseases in PsA is reportedly comparable to that observed in RA (9). A meta-analysis based on 11 studies found a 43% increase in CV diseases in PsA compared to the general population. In addition, the risk of myocardial infarction, cerebrovascular disease and heart failure was increased by 68%, 22% and 31% respectively (10). The relationship between inflammation in patients with PsA and the subsequent development of CV diseases has been examined in specific cohort studies. The conclusions indicate that markers of disease activity and/or severity (polyarthritis, dactylitis, extent of PsO, elevation of acute phase reactants) in PsA were associated with future CV events (11, 12). In addition, the extent of atherosclerotic plaque was associated with disease activity and inflammation in patients with PsA (13). In a meta-analysis including 31 studies involving 665,009 patients with PsO and 17,902,757 non-psoriatic control subjects, pooled analyses revealed that PsO patients, especially severe PsO, had a higher risk of ischemic heart disease, myocardial infarction, stroke, thromboembolism, arrhythmia and cardiovascular death. Psoriasis remained an independent risk factor for adverse CV outcomes (14). The association between venous thromboembolism (VTE) and PsD has also been reported: a systematic review and meta-analysis based on 13 cohort studies reported an increased risk of incident VTE in patients with PsO (pooled Hazard ratio (HR)[95% confidence interval (CI)]: 1.26 [1.08-1.48] but also in patients with PsA (pooled HR: 1.24 [1.01- 1.53]) (15). Furthermore, it is considered that the CV burden is higher in patients with PsA compared to patients with PsO alone: in a population-based study from Taiwan, patients with PsA had a higher incidence of cerebrovascular diseases compared to psoriatic patients without arthritis (HR: 1.83 [:1.17-2.82]) (16). A comparative study by Husted et al. found significantly higher prevalence of hypertension, obesity, dyslipidemia, type 2 diabetes and CV diseases in patients with PsA compared to those with PsO alone (unadjusted odds ratio (OR) for CV diseases : 2.59 [1.43-4.67]) (17). Finally, the risk of major adverse CV events (MACE) in patients with PsA not using a disease-modifying antirheumatic drug (DMARD) was similar to the risk observed in RA after adjustment for traditional CV risk factors (HR for PsA : 1.24 [1.03-1.49], HR for RA : 1.39 [1.28-1.5]) (18).

### Obesity

Overweight and obesity are more common in patients with PsO or PsA compared to the general population, and compared to patients with RA (19, 20). In a Canadian case-control study comparing the body mass index (BMI) of patients with PsA, PsO, RA and normal controls, the proportion of individuals with obesity was 37%, 29%, 27% and 18% respectively. The odds of obesity were 61% higher for PsA than PsO (21). This was confirmed in a cohort study from a UK database, in which the prevalence of obesity was higher in PsA compared to PsO alone (18). Some studies suggest that being overweight or obese could be a risk factor for developing PsO and/or PsA. In the prospective Nurse’s Health Study, there was a link between weight gain and incident PsO (22). The prevalence of obesity is higher in psoriatic patients than in the general population and the BMI of psoriatic patients tends to increase over time. Furthermore, being overweight with abdominal obesity and being obese is more common in children with PsO than in controls (23). Using Mendelian randomization, it was established that a higher BMI increase the odds of PsO (by 9% per 1 unit increase in BMI) (24). Moreover, it was demonstrated that obesity could be a factor for the transition from skin disease to joint involvement. Indeed, in an electronic database of medical records from the UK, the incidence of PsA increased in parallel with BMI both in patients with PsO and in the general population (25). In a prospective study performed in the USA (US Nurse’s Health Study II), BMI, weight changes and measures of central obesity were recorded over a 14-year period. It was found that BMI was monotonically associated with an increased incidence of PsA (26). In contrast, there were limited data on body composition in PsD. Using dual-energy X ray absorptiometry (DXA) measurements, Pedreira et al. found an increased total fat percentage in patients with PsA, but no changes in lean mass (27). Our group evaluated body composition in patients with PsA or PsO alone and matched controls. We found no significant differences in body composition measurements between PsA patients and their matched controls, while patients with PsO had higher visceral fat compared to their controls (28).

### Diabetes, dyslipidemia and metabolic syndrome

Several studies have shown that patients with PsD are at increased risk of diabetes (4). In a systematic review and meta-analysis of cohort studies, the incidence of type 2 diabetes was 13.4 and 7.8/1000 patient-years in patients with PsA and non-rheumatic control subjects, respectively (29). In a systematic review of CV comorbidities in PsA, the prevalence of diabetes was found to be increased in patients with PsA compared with controls (30). In addition, patients with PsA are more likely to have higher fasting glycemia compared to patients with RA (20). The prevalence of diabetes and insulin resistance were higher in 102 patients with PsA compared to 82 control subjects, after adjusting for BMI (31). In a cohort of 60 children with PsO aged 3-10 years, 27% had insulin resistance (32). Compared to mild disease, the severity of PsA (defined by joint erosive changes, osteolysis and sacroiliitis) was linked to insulin resistance in an Irish cohort study of 283 patients (OR: 3.49 [1.08- 11.2]) (33). The risk of diabetes seems to be higher in women and in patients with active disease (34). Abnormal lipid profile has been reported in PsA: indeed, patients were characterized by an unfavorable atherogenic ratio with a reduction in HDL cholesterol and elevated circulating triglyceride levels (20, 30). Systemic inflammation, as estimated by C-reactive protein (CRP) has been linked to low HDL cholesterol and high total/HDL cholesterol ratio (13). Some studies have also shown that patients with PsA had more lipid abnormalities compared to patients with PsO alone (28% versus 13.5%, OR 2.5 [95%CI 1.7- 3.3]) (35). In addition, the lipid profile is more altered in PsA compared to RA (35). Hypertension is also a well-recognized comorbidity of PsA. In the study by Husted *et al*, hypertension was the most frequent comorbidity of PsA (37.1%) and was more prevalent than in patients with PsO alone (20%) (17). Systemic inflammation seems to influence hypertension in PsA (31). Accumulating evidence suggests that there is a relationship between PsD and an increased risk of metabolic syndrome (MetS) (36). Metabolic syndrome and its components (central obesity, hypertension, insulin resistance and dyslipidemia) are strongly represented in PsA, ranging from 24% to 58% (37). MetS has consistently been reported in several series of patients with PsA, and is associated with the severity of the disease (30, 33), more consistently than in cases of PsO (38). Again, the prevalence of MetS is higher in PsA than in PsO alone (16) and higher than in RA (20). In a systematic review and meta-analysis, the pooled prevalence of MetS was found to be higher in PsA (0.46 ± 0.06 [0.40- 0.51] than in PsO (0.34 ± 0.03 [0.32-0.37]) (39).

## Pathogenic mechanisms involved in cardiometabolic diseases of PsD

**Figure 1:**
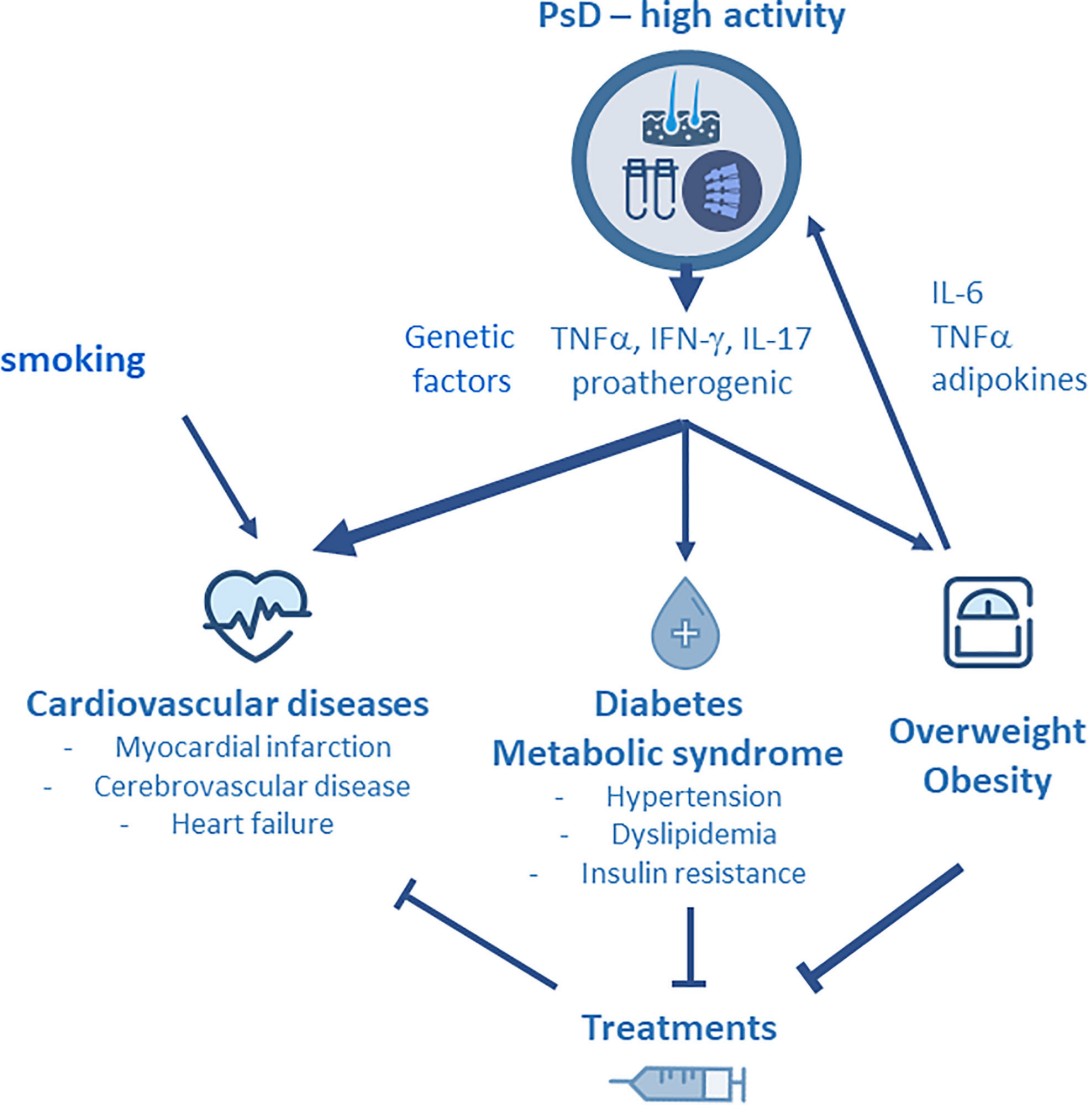
Due to disease activity, psoriatic disease (PsD) is associated with the production of Th1 (TNFα, IFNγ) and Th17 (IL-17, IL-22) derived cytokines that have potential effects on the blood vessels. They lead to endothelial activation, vascular dysfunction, altered lipid profile and prothrombotic effects, and ultimately, they promote atherosclerosis and cardiovascular (CV) disease. PsD is strongly associated with specific comorbidities including obesity and metabolic syndrome (MetS). Smoking and genetic factors may contribute to the development of CV diseases in PsD. Obesity is a predisposing factor for the developement of PsD and contributes to disease activity by the release of adipokines. Specific comorbidities, such as MetS and obesity, and therapeutic response are interrelated in PsD : on the one hand, MetS and obesity impair the therapeutic effectiveness of TNF inhibitors (TNFi), while on the other hand, methotrexate and TNFi have been shown to limit the CV burden of PsD.

It is now well established that inflammation is a major determinant of atherosclerosis, playing a role in plaque formation and progression (40, 41). The relationships between inflammation and atherosclerosis have been well demonstrated in RA: proinflammatory cytokines such as TNFα, IL-1 and IL-6 produced by various activated cells (T lymphocytes, monocytes, mastocytes, adipocytes) are released into the circulation and have potential effects on different tissues, including the blood vessels, leading to endothelial activation, vascular dysfunction as well as altered lipid profile and prothrombotic effects (6). The ultimate consequence is to promote atherogenesis. In PsD, several cytokines may play a role in atherosclerosis. Shared chronic inflammatory pathways such as Th1 and Th17 activation lead to the production of proinflammatory cytokines (TNFα, IFN-γ following Th1 differentiation, and IL-17A, IL-17F, IL-22 after Th17 activation). These inflammatory mediators are increased in the skin, the joint and the circulation in patients with PsO and/or PsA compared to subjects from the general population. They have various effects on the endothelium, leading to a proatherogenic phenotype (42). Circulating TNFα alone or in combination with IL-17 have been associated with endothelial dysfunction in PsA. It is well established that IL-17 is a key cytokine driving inflammation in PsD, and IL-17 is considered to be a solid candidate linking PsD to the developement of CV diseases (43, 44). As it was observed in RA, IL-6 is involved in joint inflammation and degradation of patients with PsA. Specific polymorphisms of *IL-6* gene have been linked to CVD in PsA (45). The development of MetS in PsD is related to the systemic and chronic inflammation. In addition, obesity, type 2 diabetes and insulin resistance share common inflammatory pathways with the involvement of specific inflammatory cytokines such as TNFα and IL-6 (44). Moreover, adipose tissue is a reservoir of proinflammatory cytokines, mainly IL-6 and TNFα, and obesity may promote expansion of Th17 cells (46). The links between obesity and inflammation may also be substantiated by the involvement of specific adipokines, such as leptin and adiponectin, in the metabolic disturbances observed in PsD (47, 48). Smoking is a risk factor for CV disease that has been found significantly associated with PsO (4) and PsA (49). In parallel, gout augments the risk of CV diseases in PsO: in a population-based cohort study from Taiwan, patients with PsO and gout had a significantly higher risk for CV disease compared to patients with PsO alone (50) (**Figure 1**). Finally, the concept of “psoriatic march” has been proposed in order to explain the link between inflammation and the development of atherosclerosis in PsD: systemic inflammation, in conjunction with obesity and metabolic abnormalities, promotes the developement of insulin resistance, endothelial dysfuction, atherosclerosis and ultimately CV diseases (51).

## Clinical implications of cardiometabolic conditions in PsD

### Screening for cardiometabolic diseases

The European Alliance of Associations for Rheumatology (EULAR) group has published recommendations for the management of the CV risk in inflammatory rheumatic diseases (IRD) including PsA (52). It is considered that the different tools assessing CV risk in IRD, such as the Framingham risk score, the European systemic coronary risk evaluation SCORE or the Reynold’s CV risk score, all underestimate the real risk in IRD, especially in RA, but also in PsA (53). Thus, the EULAR has proposed the use of a correcting factor, by multiplying the score obtained in patients with RA by 1.5. In PsD, this correcting factor has not yet been validated. Despite the substantial evidence in support of an increased CV risk in PsD, the different CV risk factors remain undertreated (54). In a population-based study from the UK, the management of CV risk factors in patients PsA was similar to that of subjects from the general population, despite an increased prevalence of hypertension, hyperlipidemia, diabetes mellitus and obesity (35). Despite a vast body of literature showing an overall increased risk of CV disease in patients with PsO, it has been found, that patients with psoriasis and CV risk factors receive less cardio-protective medical therapy than controls without PsO, or no such treatment at all (55). The Joint American Academy of Dermatology — National Psoriasis Foundation guidelines (56) advocate that dermatologists inform psoriatic patients of their elevated CV risk and ensure engagement with their primary care doctor or cardiologist. The US (56) and European Academy of Dermatology and Venerology’s (57) guidelines recommend screening patients with PsO upon systemic treatment for CV disease risk factors every twelve months. Furthermore, the British (58) and French (59) guideline recommends CVD risk assessment in adults with severe PsO at presentation and further CV assessment every five years (58). The European Society of Cardiology (60) recommends CV screening and similar therapeutic interventions as in the general high-risk population.

These data strongly underline the need to carefully screen for and manage the different CV risk factors in PsD. In parallel to CV risk factors, obesity, diabetes and MetS require specific attention in patients with PsD to be adequately managed (7, 37, 43). Optimal lifestyle modification in PsO including hypocaloric and Mediterranean diet and smoking cessation if required, is a cornerstone of strategies to reduce CV disease (61). Clinical trials evaluating traditional CV risk factor treatment thresholds and goals in the psoriasis population are lacking. However, given the pattern of dyslipidemia, lipid-lowering, i.e. statins, play a key role in CV risk reduction strategies in PsO (62). While it is also reasonable to promote aggressive blood pressure and hemoglobin A1c goals, in-line with ACC/AHA recommendations in patients at elevated risk of CVD, clinical studies evaluating this approach in PsO are still needed (63). Whether statin therapy in PsO confers additional anti-inflammatory benefit beyond lipid-lowering is not yet known. Aspirin in the primary prevention of CVD is controversial even in the non–psoriatic patient (64).

### Impact of metabolic diseases of PsD on the *therapeutic response to targeted treatments*

Obesity and MetS are associated with impaired response to the treatments that are used in PsD. In contrast, weight loss has a positive impact on the severity of PsO (65). Overweight and obesity have been reported to be associated with a reduced probability of achieving a sustained minimal disease activity (MDA) state in PsA, irrespective of the treatment used (–) (66). In an Italian prospective study, being obese was an independent risk factor for not acheiving MDA (HR: 4.9 [3.04- 7.87]) and for relapse over 24 months (67). The response to TNF inhibitors (TNFi) is particularly influenced by BMI: in a meta-analysis including 22 cohorts with PsD, obesity was associated with poor response to TNFi in obese patients (OR for failing to respond to TNFi: 1.57 [1.30-1.89]) (68). MetS was also associated with poor response to TNFi: in an Italian cohort of patients with PsA receiving their first TNFi, the presence of MetS was associated with a lower probablility of achieving MDA at 24 months (OR: 0.56) (69). Lastly, in the Danish (DANBIO) and Icelandic (ICEBIO) biologics registries, obesity was associated with an increased risk of TNFi withdrawal (HR: 1.6 [1.3-2.0]) and reduced EULAR good or moderate responses (OR: 0.47 [0.29- 0.72]) (70). For the IL-12/23 inhibitor ustekinumab, data showed that obesity impared the skin response (71). For secukinumab, BMI does not seem to influence the therapeutic response in PsA according to recent data (72). Tofacitinib is a pan-JAK inhibitor (JAKi) licensed for the treatment of PsA. In a *post hoc* analysis of 3 randomized controlled trials, American College of Rheumatology (ACR) response rate to tofacitinib was reduced in patients with BMI ≥ 35 kg/m² (73).

### Impact of systemic treatments on cardiometabolic diseases of PsD

Taking into account the link between systemic inflammation and CV diseases, it is conceivable that controlling inflammation by systemic agents may positively impact the CV risk in PsD (73, 74). It is now well demonstrated that conventional synthetic agents such as methotrexate (MTX), but also TNFi, improve the CV burden of patients with RA (75). In PsD, MTX has been associated with a reduction in CV risk (76). A meta-analysis examined the effects of MTX on CV risk in patients with RA, PsO or PsA. The conclusion was that MTX was associated with a 21% reduction in overall CV risk and an 18% reduction in the risk of myocardial infarction (77). In a population-based cohort study in the USA, Ogdie et al. concluded that the risk of MACE was higher in patients with PsA or PsO not receiving a conventional synthetic DMARD (csDMARD) or in patients with severe PsO (18). A larger body of data is available regarding the specific impact of TNFi on CV events. Their beneficial effects are well demonstrated in RA. The meta-analysis by Roubille et al. based on 6 studies in PsD showed a significant reduction in CV events under csDMARD or TNFi in PsO and PsA (relative risk [RR] : 0.72 [0.57-0.91] and 0.7 [0.54- 0.9], respectively) (75). A second meta-analysis came to the same conclusion, namely that compared to MTX, the risk of CV events was markedly decreased under TNFi (RR: 0.67 [0.52-0.88]) (78). Data regarding other bDMARDs are more limited. Conversely, the impact of PsD treatment on CV risk must be discussed. Indeed, blockade of the IL-23/IL-17 axis, however, warrants caution as a cardiovascular intervention. A case-time-control analysis based on data from 9290 patients with records in the French national health insurance database from 2010 to 2016 suggested that the initiation of ustekinumab treatment is associated with an increased risk of acute coronary syndrome or stroke in patients with a high baseline cardiovascular risk (79). IL-17A appears to be a differential regulator of atherosclerosis, and its effects in mouse models suggest that its modulation may have contradictory effects on plaque size and possibly stability. Targeting this pathway has improved PsO, but may augment CV risk in certain patients (80, 81). However, it has been suggested in an observational study of Pso patients that secukinumab, an IL-17 inhibitor, improved left venricular function and coronary flow reserve compared with MTX and cyclosporine (82). In the same way, concerns have been raised about the risk of MACE under briakinumab, another anti-IL-23 agent. However, a meta-analysis of 38 randomized clinical trials in PsO concluded that there was no increase in MACE with the use of different biological agents, including ustekinumab but also TNFi and anti IL-17 agents (83). A large Korean PsO cohort study assessed MACE risk according to the treatment modalities of phototherapy, biologic and conventional systemic agents. Phototherapy and biologic groups showed a lower incidence of MACE than the control cohort, and the difference in the cumulative incidence remained significant over the 36-month follow-up period. Cyclosporine and mixed conventional systemic treatments were significantly associated with an increased MACE risk, whereas MTX was not associated with MACE (84). Lastly, concerns have recently emerged regarding the risk of thrombosis under the JAKi tofacitinib, leading to specific warnings. The ORAL Surveillance randomized trial analyzed the safety of tofacitinib (5 and 10 mg twice daily) versus a TNFi in subjects with RA aged 50 years or older who had at least one additional CV risk factor (85). The study concluded that there was a higher risk of MACE and malignancies with tofacitinib as compared to TNFi in patients with RA (HR for MACE: 1.33 [0.91-1.94]; HR for malignancies: 1.48 [1.04- 2.09]). Following these results, healthcare professionals were advised to consider the benefits and risks of tofacitinib, but also other JAKi, when deciding to prescribe and continue patients on the drug. However, in the interim analysis of tofacitinib in PsA patients in the OPAL Balance trial (3 year, open-label extension study of tofacitinib in PsA), there was no evidence of an increased risk of CV events (86). Similarly, in an integrated analysis of 2 randomized, placebo-controlled phase 3 trials with upadacitinib in PsA, including one trial with adalimumab, rates of MACE were similar across treatment groups (87). It has been reported that TNFi induce body composition changes in IRD with weight and fat mass gain, especially in the central abdominal region (88). In contrast, there are some data showing that TNFi may improve different components of MetS (89) and diabetes (90) in PsD (91).

## Conclusion

PsD is associated with a higher prevalence of cardiometabolic diseases compared to the general population. This prevalence is higher in PsA compared to PsO alone, and also higher compared to other IRD, such as RA. Obesity and MetS are strongly represented in PsD, and obesity is a known risk factor for the development of PsO and PsA. These metabolic comorbidities must be adequately screened for and managed by the physicians caring for patients with PsD. Obesity influences the treatment response to specific biologic agents such as TNFi. In parallel, MTX and TNFi have been shown to have a positive impact on CV risk and on MetS.

## Author contributions

ET performed the bibliographic search and wrote the first version of the manuscript. FA completed the bibliographic search and specific sections of the manuscript. IG-S made the figure. All the authors reviewed and edited the manuscript.

## Conflict of interest

The authors declare that the research was conducted in the absence of any commercial or financial relationships that could be construed as a potential conflict of interest.

## Publisher’s Note

All claims expressed in this article are solely those of the authors and do not necessarily represent those of their affiliated organizations, or those of the publisher, the editors and the reviewers. Any product that may be evaluated in this article, or claim that may be made by its manufacturer, is not guaranteed or endorsed by the publisher.
